# Herbal Plants: The Role of AhR in Mediating Immunomodulation

**DOI:** 10.3389/fimmu.2021.697663

**Published:** 2021-06-24

**Authors:** Izzah Bungsu, Nurolaini Kifli, Siti Rohaiza Ahmad, Hazim Ghani, Anne Catherine Cunningham

**Affiliations:** Pengiran Anak Puteri Rashidah Sa’adatul Bolkiah (PAPRSB), Institute of Health Sciences, Universiti Brunei Darussalam, Bandar Seri Begawan, Brunei

**Keywords:** AhR, flavonoids, immune response, immunomodulatory, herbal plants

## Abstract

The prevalence of chronic inflammatory diseases including inflammatory bowel disease (IBD), autoimmunity and cancer have increased in recent years. Herbal-based compounds such as flavonoids have been demonstrated to contribute to the modulation of these diseases although understanding their mechanism of action remains limited. Flavonoids are able to interact with cellular immune components in a distinct way and influence immune responses at a molecular level. In this mini review, we highlight recent progress in our understanding of the modulation of immune responses by the aryl hydrocarbon receptor (AhR), a ligand-dependent transcription factor whose activity can be regulated by diverse molecules including flavonoids. We focus on the role of AhR in integrating signals from flavonoids to modulate inflammatory responses using *in vitro* and experimental animal models. We also summarize the limitations of these studies. Medicinal herbs have been widely used to treat inflammatory disorders and may offer a valuable therapeutic strategy to treat aberrant inflammatory responses by modulation of the AhR pathway.

## Introduction

Aberrant activation of immune responses is an underlying cause for the development of chronic inflammatory diseases which either excessively activate immune cells that contribute to tissue damage or suppress immune cells and enable cancer cell proliferation and metastasis (1). Non-toxic herbal compounds such as flavonoids have been shown to induce protective effects against multiple chronic inflammatory diseases (2) including IBD (3), autoimmunity (4, 5) and cancer (6, 7). Understanding the molecular mechanisms of flavonoids and their potential pathways is crucial to identify therapeutic targets for more effective and safer interventions for inflammatory diseases.

Flavonoids are polyphenols, acting as the main bioactive metabolites in various plants, which contribute to the color, taste as well as pharmacological and biochemical effects (8). Good sources of flavonoids include plant-derived food such as fruits, vegetables, tea, cocoa products, nuts, legumes, and herbal plants (8).

**Figure 1:**
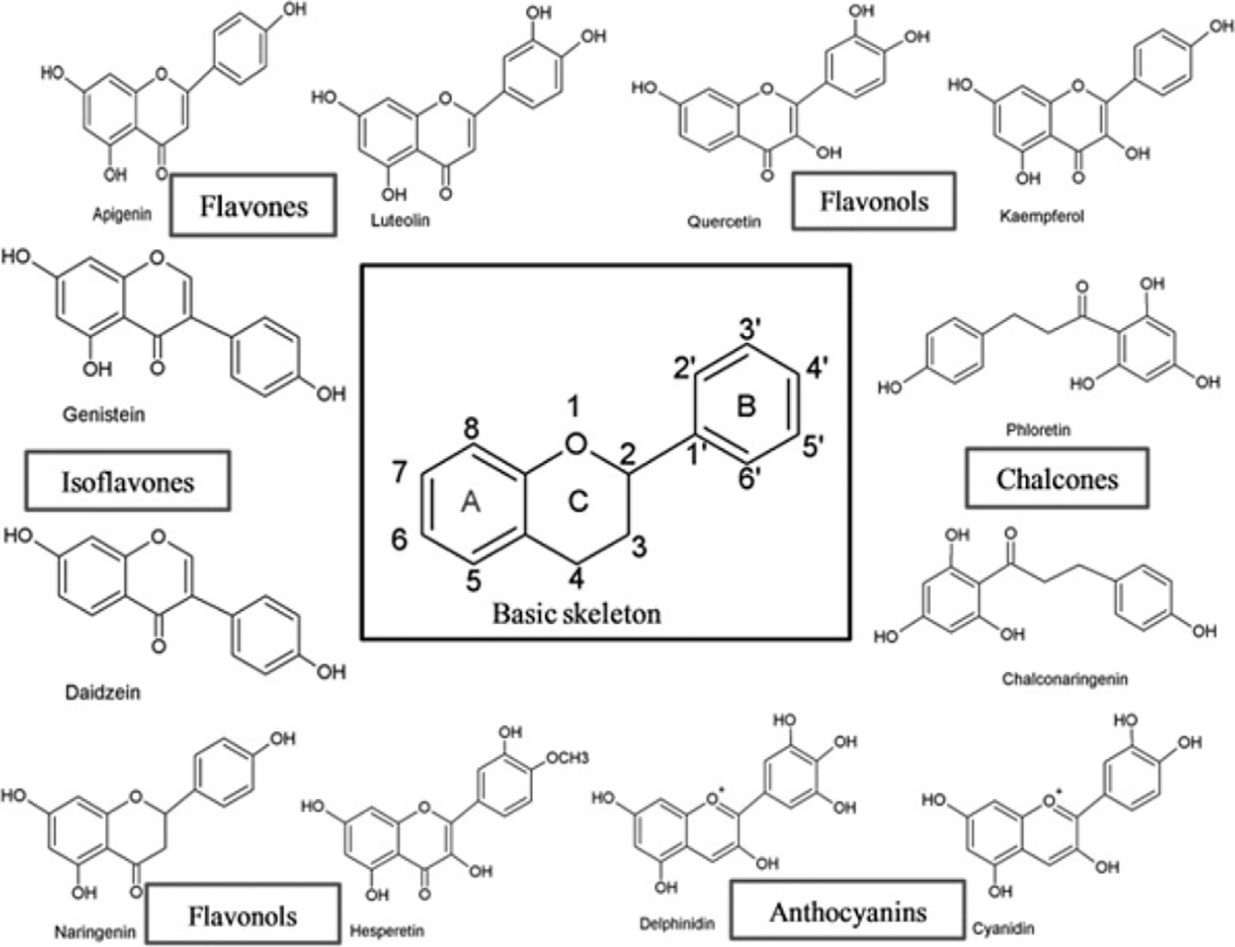
The basic skeleton structure of flavonoids and six major classes of flavonoids (9).

Flavonoids have a broad range of structures depending on the position of the carbon in the C ring to which the B ring is attached, and the degree of saturation and oxidation of the C ring (**Figure 1**). Flavonoids can be categorized into six major groups from a structural standpoint, namely flavonols, flavones, isoflavones, flavonones, flavanols and anthocyanidins (9).

Numerous *in vitro* and *in vivo* studies have demonstrated the health-promoting effects flavonoids possess including antioxidant, anti-inflammatory and immunomodulatory properties (2, 9). The mode of action of flavonoids include their ability to directly interact with immune cells, modify the production of cytokines and inhibit inflammatory signaling pathways (10). For example, fisetin (a flavonol) was found to inhibit maturation and activation of dendritic cells and inhibit Th1 polarization by significantly inhibiting the expression levels of costimulatory proteins CD40, CD80, CD86 and MHC Class II in a dose-dependent manner (11). Similar effects have been observed in dendritic cells harvested from mouse bone marrow using quercetin (another flavonol) (12). In a murine model of experimental autoimmune thyroiditis (EAT), luteolin (a flavone) led to decreased lymphocyte infiltration by inhibiting interferon-γ-induced COX-2 and pro-inflammatory cytokine tumor necrosis factor-α (TNF-α) (13). Apigenin (a flavone) has been demonstrated to decrease colonic damage scores and colonic weight/length ratio in a rat model of colitis (14). Oral administration of apigenin resulted in normalization of some colonic inflammatory markers such as TNF-α, transforming growth factor β (TGF-β) and interleukin-6 (IL-6) (14). Genistein (an isoflavone), kaempferol, quercetin (a flavonol), and daidzein (an isoflavone) were shown to inhibit STAT-1 and NF-κB activation (15). Interestingly, flavonoid metabolism in the gut also modulates intestinal immune responses *via* the activation of T-cell differentiation, gut microbiota alteration and cytokine production (16). This highlights the importance of dietary interventions containing flavonoids to combat human chronic diseases (16). However, less is known about how flavonoids regulate immune components and immune signaling pathways at a molecular level. Structural studies reveal that flavonoids are ligands of the aryl hydrocarbon receptor (AhR) (17–19).

## Aryl Hydrocarbon Receptor (AhR)

AhR is a ligand-dependent transcription activator that responds to a variety of molecules from the environment including dietary, metabolic products and pollutants (20). This protein was first identified as the dioxin receptor that mediates biotransformation and elimination of harmful xenobiotics, it’s activation leading to toxicity and tumor development (21, 22). AhR is increasingly recognized as an important immune modulator implicated in many chronic inflammatory diseases (23).

The inactive form of AhR resides in the cytoplasm, complexed with several chaperone proteins such as heat-shock protein (HSP90), p23 (17), c-SRC and AhR interacting protein (AIP) (20, 24, 25). Upon binding with environmental ligands, AhR translocates to the nucleus which leads to heterodimerization with the AhR nuclear translocator (ARNT). AhR-ARNT complex binds to the xenobiotic response element (XRE) in regulatory genes to induce specific transcription of gene expression including drug and ligand-metabolizing enzymes such as CYP1A1, CYP1A2 and CYP1B1 and AhR repressor (AhRR). This pathway is tightly controlled through proteasomal degradation of AhR ligand metabolism by CYP1A1, and AhR/ARNT complex disruption by AhRR (26).

AhR can also interact with other transcriptional regulators including retinoblastoma protein (Rb), NF-kB, the estrogen receptor and modulate their activity (27). AhR interaction with Rb leads to cell cycle arrest in the G1-phase in several cell lines (28), while AhR interaction with NF-κB induces the expression of cytokines and chemokines such as B-cell activating factor of TNF family (BAFF), CXCL13, CCL1 and the transcription factor interferon responsive factor 3 (IRF3) (29). It has been shown that 2,3,8,7-tetradichlorobenzo-p-dioxin (TCDD)-mediated AhR activation led to its recruitment to a non-consensus XRE, ie E2F-regulated-S phase loci (30). Kruppel-like factor 6 (KLF6) is another AhR-DNA binding partner recently reported (31). KLF6 is a tumor suppressor and it’s mutations have been associated with a number of cancers such as hepatocellular carcinoma, gastric and colon cancers (31). KLF6 activates p21^cip1^ *via* an AhR-dependent mechanism which leads to the inhibition of cell cycle progression (32). In addition, AhR has also been demonstrated to control biological processes through the regulation of retrotransposons, micro-RNAs and long non-coding RNAs which are known to regulate multiple target genes (33).

AhR can influence chromatin architecture by interacting with Brahma/SWI2-related gene 1 (Brg1) subunit of the SWI/SNF chromatin-remodeling complex (34). AhR can also affect local histone hyperacetylation and methylation either by directly interacting with coactivators such as the steroid receptor coactivator-1 (SRC-1) complex (35) or by displacing histone deacetylase (HDAC) complexes (36).

There have also been reports that show AhR can act independently of ligand activation under certain conditions (37, 38). However, the physiological relevance of these observations remain to be evaluated. Overall, activation of AhR by a ligand can induce non-genomic and genomic pathways that promote transcriptional events and modulation of myriad biological processes including immune responses. Through both mechanisms, AhR targets specific gene expressions associated with inflammation including NF-κB, immune regulatory and growth factors (20). AhR is also able to control the differentiation of several cell types in the immune system including innate dendritic cells (DCs), macrophages and natural killer cells (39) and adaptive B and T cells relevant to inflammation (40–45).

## AhR Expression

AhR is widely expressed throughout the body particularly in the liver, placenta (46) and in epithelial barriers such as the skin, gut and lung mucosa (47). AhR is also highly expressed by multiple cell types at these barrier sites including intestinal epithelial cells (IECs) (48), intraepithelial lymphocytes (IELs) (49), innate lymphoid cells (ILCs) (50) and intraepithelial CD8αα-expressing lymphocytes (49). AhR levels are very low in naïve T and B cells, Th1 and Th2 helper T cells, moderate in natural killer (NK) cells but very high in Tregs, Th17 cells (24, 25, 39, 47), B cells (51, 52) and DCs (53–57). AhR expression is also pronounced in unconventional peripheral γδ T cell subsets such as TCRγ1, TCRγ2, TCRγ3, TCRγ4, TCRγ5 and TCRγ6 (58). Systemic Vγ5-expressing γδ T cells produce IL-22 in response to AhR activation (59) while epidermal Vγ3 and intestinal Vγ5-expressing γδ T cells require AhR for survival as studies in AhR-deficient mice showed they were lacking these subsets (26). Vγ4-expressing γδ T cells which are predominant in the lungs, reproductive tract and oral mucosa express very high levels of AhR. However, these cells are not reduced in AhR deficient mice suggesting they may also have different roles (26). Overall, AhR has become a key player in maintaining tissue integrity, tissue repair and immune protection against environmental challenges, particularly at epithelial barrier sites.

## AhR Ligands

Numerous AhR ligands have been identified which consist of xenobiotic compounds and natural compounds that are derived from food and host/microbiome metabolism (17, 18). Many of these ligands have been shown to impact on immune responses *via* modulation of immune cell function and differentiation.

The first prototypical AhR ligand studied was TCDD (21), an environmental contaminant that has intrigued toxicologists for decades. Early studies mostly focused on the immune toxicity and carcinogenic effects of TCDD in humans and animal models. It has profound immunosuppressive effects that are undesirable as it increases susceptibility to bacterial and viral infections and tumor growth (60). Other reported effects include thymic involution, depletion of lymphoid organs, thymocyte and T cell apoptosis (26, 61). However, during inappropriate immune responses, the effects of AhR activation by TCDD seem beneficial for preventing development of diseases such as allograft rejection, allergic responses, autoimmunity including type 1 diabetes (60). TCDD has been shown to suppress Th1, Th2 and Th17-cell mediated responses and promotes the development of Tregs by a TGF-β-dependent mechanism (25, 41, 60).

Tryptophan (Trp) amino acid metabolism is an emerging key family of AhR ligands. Degradation of Trp *via* enzymes including indoleamine 2,3-dioxygenase (IDO) and tryptohan 2,3-dioxygenase (TDO), photo-oxidation and bacterial degradation generate distinct AhR agonist ligands such as kynurenine (Kyn), 6-formylindolo[2,2-b]carbazole (FICZ) and indoles respectively. It has been reported that Kyn is produced in glioma cells and has been shown to promote the differentiation of Tregs and promote immunosuppression in the tumor microenvironment (62). Photo-oxidation of Trp to FICZ has been shown to impact immune responses in an experimental autoimmune encephalitis (EAE) mouse model (24). The activation of AhR by FICZ interfered with Treg cell development, boosted Th17 cell differentiation and increased the severity of EAE in mice (41). Interestingly, AhR activation by FICZ also strongly promotes expression of IL-22 (63, 64) which is a member of the IL-10 family of cytokines. Although IL-22 has been shown to be pro-inflammatory and can induce skin inflammation (65, 66), it has also been reported to prevent tissue damage and aids in repair of the gastrointestinal tract. Bacterial degradation of Trp to produce indole metabolites such as indole-3-aldehyde (IAld) has been demonstrated by *Lactobacillus* species in the intestine (67). Zelante et al. demonstrated that activation of AhR by lald helps maintain intestinal homeostasis and prevents colonization by pathogenic microorganisms such as *Candida albicans* and inhibits development of inflammatory disorders such as IBD and cancer (67). Deficits in commensal bacteria producing Trp-derived AhR agonist may contribute to the pathogenesis of human IBD (68).

Dietary factors can also be a source of AhR ligands. Vegetables such as broccoli, cauliflower, Brussel sprouts and cabbages contain indole-based glucobrassicin (24, 26) which can be converted into AhR agonist precursors such as indole-3-carbinol (I3C) and indole-3-acetonitrile (I3ACN) by chewing. I3C and I3ACN can be further converted into AhR activating metabolites such as 3,3’-di-indolyl-methane (DIM), [2-(indol-3-ylmethyl)-indol-3-yl] indol-3-ylmethane (LTr1), and indolo[3,4-b] carbazole (ICZ) (69). These dietary compounds have been shown to promote maintenance of the intraepithelial lymphocytes (IELs) and innate lymphoid cells (ILCs) allowing cell proliferation, immune surveillance and modulation of the gut inflammation (69). Other therapeutic effects of I3C and its precursors reported include modulation of inflammation in experimental animal models of multiple sclerosis (70) and other murine models (48) *via* AhR-dependent induction of FoxP3 regulatory T cells.

Natural compounds from plants have also been reported to be ligands for AhR. Indirubin and indigo are phytochemicals with mild AhR agonistic activity. Their concentrations may be too low to be considered as relevant physiological ligands, however their dietary accumulation may have the potential to affect AhR activation and contribute to the maintenance of mucosal integrity in the gastrointestinal tract (24). A study by Kawai et al. on murine dextran sulfate-induced colitis showed increased mRNA expressions of IL-10 and IL-22 following indigo treatment derived from herbal plants (71). The group also showed the expansion of IL-10 producing CD4+ T cells and IL-22 producing CD3-RORγt cells, but interestingly not CD4+FoxP3+ regulatory T cells in C57BL/6J mice (71). Indigo is a potent inducer of IL-10 and IL-22 that protects against high-fat diet (HFD)-induced insulin resistance in a C57BL/6J diet-induced obesity murine model that is linked to a reduction in harmful inflammatory immune cell accumulation in the intestine, visceral adipose tissue and liver (72).

Flavonoids are another group of phytochemicals that are thought to be important ligands of AhR. They have been demonstrated to confer protective effects in a range of AhR-dependent *in vitro* and *in vivo* models associated with ulcerative colitis, allergy and cancer. Flavonoids have been shown to control inflammatory responses by inhibiting certain inflammatory pathways, downregulating pro-inflammatory cytokines and promoting tolerogenic immune responses by AhR dependent mechanisms (**Table 1**). Activation of AhR by a non-toxic flavonoid, β-naphthoflavone (βNF) was observed to reduce the severity of colitis in a murine model through inhibition of NF-κB pathway and pro-inflammatory cascade of cytokines (73). Activation of AhR by cardamonin (a flavone) isolated from *Alpinia katsumadai* Hayata seeds, significantly improved the condition of male C57BL/6J and BALB/c mice through inhibition of NLRP3 inflammasome activation (74). Alpinetin (a flavone) also isolated from *Alpinia katsumadai* Hayata seeds alleviated colitis through restoring Th17/Treg balance in the colon *via* activation of AhR. *In vitro* assays showed alpinetin induction of Treg differentiation but less effect was observed on Th17 differentiation in this study (75). Activation of AhR by naringenin (a flavonone) commonly found in grapefruit promoted the differentiation of Tregs in murine models of allergy (76). Interestingly, AhR also contributes to flavonoid metabolism and this will affect bioavailability *in vivo*. AhR activation upregulates gene expression of xenobiotic enzymes creating positive feedback loops. The xenobiotic enzymes can be distinguished into phase 1 and phase II enzymes. Phase 1 enzymes mediate oxidation, reduction and hydrolysis reactions, and flavonoid metabolism occurs mainly in the intestine (77). The cytochrome P450 (CYP450) family are crucial to phase 1 biotransformation of flavonoids. Once flavonoids enter intestinal epithelial cells, phase II enzymes produce the corresponding conjugated metabolites. Three types of phase II enzymes are reported to metabolize flavonoids, uridine-5ʹ-diphosphate-glucuronosyltransferases (UGT), sulfotransferases (SULT), and catechol-O-methyltransferases (COMT) (78, 79). Phase II conjugation also occurs in the liver where flavonoids can be further conjugated (e.g., sulfation/methylation) (79) then transported to the portal vein or lymphatics. In addition, the gut microbiota also contribute to flavonoid metabolism (80) and may compensate for phase 1 and phase II enyzmes (79).

**Table 1:** Effect of flavonoids-induced immunomodulation via AhR.

Flavonoid	Type of study	Disease involved	Mechanism	References
βNF	*In vivo*	Colitis	Inhibition of NF-κB pathway Inhibition of pro-inflammatory cytokines TNF-α, IL-6 and IL-12	(73)
Cardamonin	*In vitro* *In vivo*	Cell line (THP-1) Colitis	Inhibition of NLRP3 inflammasome activation Inhibition of pro-inflammatory cytokines such as IL-1β, TNF-α	(74)
Alpinetin	*In vivo* *In vitro*	Colitis Cell isolation (colonic lamina propria)	Restoration of Th17/Treg balance Induce differentiation of Treg and less effect on Th17 differentiation	(75)
Naringenin	*In vivo*	Allergy	Induced Treg differentiation	(76)

The role of AhR in cancer remains unclear. Many investigators speculate AhR is a double-edged sword that can either act as a tumor suppressor or promoter. Flavonoids exhibit AhR agonist or antagonist activity in a cell line- and species-specific manner unlike TCDD (81, 82). Apigenin (a flavone), baicalein (a flavone), chrysin (a flavone), diosmetin (a flavone) and quercetin (a flavonol) are shown to activate the AhR (19) while keampferol (a flavonol), galangin (a flavonol) and naringenin (a flavanone) are demonstrated to antagonise the AhR and exhibit anti-cancer effects (83). Some flavonoids demonstrate dual AhR activity which further emphasizes the complexity of AhR responses in *in vitro* models. Opitz et al. demonstrated the pathophysiological role of AhR as a transducer of anti-tumor responses through activation by kynurenine (Kyn), a tryptophan (Trp) metabolite (84) which are both potent AhR agonists. However, emerging evidence reveals a role for AhR in halting malignant transformation and development of colorectal cancer (CRC). Metidji et al. demonstrated that application of AhR dietary ligand (I3C) can restore the Wnt-β-catenin signaling balance. The Wnt-β-catenin pathway is responsible for regulation of cell fate, proliferation, differentiation during developmental stages and tissue homeostasis (85). Deregulation of this signaling pathway has been strongly linked to many types of cancers (85) including CRC (48). In non-AhR studies, Wnt/- β-catenin signaling has been affected by flavonoids such as quercetin and fisetin, apigenin and epigallocatechin gallate (a flavanol) (85). Overall, these reports suggest that flavonoid-induced immunomodulation may also have the potential to halt tumorigenesis in an AhR-dependent manner.

### Limitations

Flavonoids are one group AhR ligands that have been shown to have therapeutic effects in various chronic inflammatory diseases. Therefore, further studies on the exact molecular mechanisms of action of flavonoids *via* AhR signaling is necessary to uncover the potential roles of AhR as an immune modulator. The majority of studies have been performed in immortalized cell lines and animal models which do not necessarily reflect immune responses by primary human cells/tissues (26). There is also evidence that flavonoids are selective AhR modulators which exhibit their agonist and antagonist activities and different potencies in tissue/organ/species-specific manner (47, 86). Therefore, it is difficult to predict their response selectivity on AhR as agonist or antagonist. Other factors which might limit the widespread acceptance of flavonoids as therapeutic agents include poor oral bioavailability and water solubility (87). Flavonoids generally have short half-lives in the human body. Hence, dietary intake of these flavonoids should be as regular as possible to maintain plasma concentrations sufficient to exert certain biological activities (87). Additionally, flavonoids can be recovered in large amounts using solvent extracts such as ethanol, methanol and acetone compared to water due to the different chemical characteristics and polarities (88). However, not all solvents are safe for consumption (89). Thus, various approaches have been taken into consideration, including application of novel drug delivery system such as nanoparticles and liposomes (87) that may help improve the bioavailability and solubility of flavonoids in human studies and unveil the full potential of these AhR ligands that can properly manipulate AhR signaling and improve disease outcomes. Curcumin, a phytochemical derived from *Curcuma Longa* has been extensively studied in nanoparticle drug delivery systems. Solid lipid nanoparticles (SLNPs) have been shown to improve the bioavailability, photostability, prevent degradation of curcumin plus target delivery to the tissue/cell of interest (90, 91). Encapsulation of curcumin by liposomes in rats demonstrated high bioavailability and more effective absorption compared to natural curcumin (92).

Herbal plants are a rich source of flavonoids. Celery, parsley, chamomile, mint and ginkgo biloba are herbs that contain high amounts of flavone (9). With modern scientific approaches, we could expand our scientific understanding of the medicinal effects of herbal plants at a molecular level considering their long history of usage and application as natural remedies for many diseases (93, 94).

## Concluding Remarks

AhR responds to natural flavonoids *in vivo* and *in vitro* which can impact immune cell function and activation. Recent studies have suggested that AhR can control inflammatory responses and modulate the differentiation of multiple immune cells implicated in inflammatory diseases. Importantly, AhR offers a unique therapeutic opportunity for wide ranging chemical structures found in herbal medicine that may activate different immunomodulatory downstream pathways. We believe that the proper manipulation of AhR signaling from plant-based products could be the next promising strategy for treatment of many inflammatory diseases.

## Author Contributions

AC conceived the idea and provided input in mucosal immunology. NK provided the pharmacological input. RA provided the nutritional input. HG provided immunological input. IB wrote the draft which was reviewed by all authors. All authors contributed to the article and approved the submitted version.

## Funding

AC is in receipt of a grant from the Herbal Research Group on In vitro evaluation of aryl hydrocarbon ligands in local plants (Grant Ref No. UBD/RSCH/URC/NIG/1.0/2019/005) – potential immunomodulatory effects for $22,000 (Brunei dollars) from 1st August 2019 to 31st July 2022.

## Conflict of Interest

The authors declare that the research was conducted in the absence of any commercial or financial relationships that could be construed as a potential conflict of interest.
